# Supplementary material for: Informing the development of diagnostic criteria for differential diagnosis of alcohol-related cognitive impairment (ARCI) among heavy drinkers: A systematic scoping review
Source: PLoS One. 2023 Feb 8;18(2):e0280749. doi: 10.1371/journal.pone.0280749 (PMC9907814; doi:10.1371/journal.pone.0280749)
Supplement: S1 Protocol — (PDF) [file pone.0280749.s001.pdf]

S1 Final protocol (October 2019) [revised December 2021]

Diagnostic criteria for alcohol-related brain injury (ARBI): protocol for a systematic scoping review to guide the development of a consensus definition

Background

Alcohol-Related Brain Injury (ARBI) is an umbrella term used for a number of neuropsychiatric conditions caused by heavy drinking. However, the diagnostic landscape and associated nomenclature for ARBI are varied, with neither the ICD nor the DSM diagnostic systems providing unified diagnostic criteria for ARBI. This systematic review will contribute to the development of a consensus definition for ARBI and support further work to explore which available cognitive assessment tool is most appropriate for accurately diagnosing patients with ARBI.

Aims

Based on a systematic scoping review of published studies and clinical guidelines, the work will aim to review differences in the clinical criteria for the definition of ARBI.

Review methods

We will use systematic review reporting guidelines (PRISMA and PRISMA-ScR) to guide the conduct and reporting of the review.

Inclusion criteria

English-language articles reporting on clinical criteria for a definition of ARBI will be eligible for inclusion. We will include articles published since 1990 that report on modifications to existing criteria or that present new criteria (including original research and consensus/statement articles), as well as those that refer to, discuss or compare existing definitions of ARBI.

Exclusions: Articles solely about the treatment and management of ARBI. Studies that solely use the ICD or DSM diagnostic systems.

Researchers have used ARBI as both an umbrella term for a range of specific disorders (namely, Wernicke-Korsakoff's Syndrome, alcohol-related dementia, and alcohol amnesic syndrome) or in reference to a spectrum of disorders (including those previously named). This systematic review will consider ARBI in its broadest sense and include articles following either application of the terminology.

We will screen the results of the search strategy to identify relevant studies in two stages. Firstly, two reviewers will double screen 20% of the titles and abstracts identified for potential inclusion. Following this round of screening, we will establish levels of agreement between the two reviewers. Any discrepancies in study selection will be identified and resolved, and if required, the inclusion criteria will be more clearly defined. A single reviewer (from the team of two) will screen the remaining titles and abstracts. Secondly, we will obtain full-text publications of any potentially relevant titles and assess them against the inclusion criteria. Two reviewers will double screen all potentially relevant articles.

Revised inclusion criteria: We will broaden our inclusion criteria to include studies about the screening, diagnosis or assessment of ARBI or cognitive impairment among patients with AUD in acute, secondary or community 'point-of-care' settings will also be eligible for inclusion.

Searching

A database of English-language articles will be compiled in EndNote based on systematic searches of the literature. As variant terms have emerged to describe ARBI we will develop an initial targeted search strategy to identify key index papers to further refine and develop terms for the search strategy.

Index searches

We will develop a targeted search strategy by combining keyword terms for alcohol and brain injury, with terms for screening, diagnosis and assessment.

Example search terms include:

Alcohol: alcoholi*, alcohol dependen*, alcohol excess* , alcohol use disorder*", alcohol misuse, alcohol addiction, Alcohol-related disorders [MeSH], Alcoholism [MeSH], Ethanol/toxicity [MeSH]

Brain injury: cognitive, neurological, neuropsychological, neurocognitive, neuropsychiatric, impair*, deficit*, disorder*, dysfunction*, defect, Cognitive Dysfunction [MeSH], Cognition Disorders [MeSH] Neurocognitive Disorders [MeSH], Korsakoff Syndrome [MeSH], Brain Diseases [MeSH]

Combined terms: alcohol-related brain injury, alcohol-related brain damage, alcohol-related brain impairment, alcohol-related cognitive impairment, alcohol-related dementia, Alcohol Amnestic Disorder [MeSH], Alcoholic Korsakoff Syndrome [MeSH]

Diagnosis: screen*, diagnos*, assess*, differentiat*, discriminat*, determinin*, distinguish*, confirmat*, ascertain*, detect*, characteris*, characteriz*, identif*, definition, define*, tool*, score*, criteri*, classification, classify, "clinical feature*", "clinical presentation*", "diagnostic feature*", validat*, reliability, accura*, specificity, sensitivity, reproducibility, predictive value, algorithm*, prevalence, significance, DSM*, ICD*, Diagnosis [MeSH]

Full searches

We will identify relevant literature through searches of electronic sources (Medline, PsycINFO, Cinahl and Web of Science), searches of Google Scholar for grey literature, relevant websites (e.g. Alcohol Change UK, Public Health England, Scottish Executive), manual screening of reference lists, and forwards and backwards citation searching (in Scopus).

Data extraction & quality assessment

We will use a coding strategy to concisely record information from the included literature. Data extraction will include the following details [* indicates revisions]:

Bibliographic details (author, year)

Study aim

Patient characteristics

- Eligibility criteria
- Setting
- Country
- Number of patients
- Details about the comparator
- Study exclusions
- Gender

- Age
- Alcohol use history*
- Global cognitive function*

Procedures and analysis

- Diagnostic/screening criteria
- Details of cognitive assessment(s)*
- Details of neuropsychological battery*
- Period of abstinence*
- Analysis*

Outcomes*

- Between group comparisons*
- Validity*
- Sensitivity and specificity*
- Other*

We expect to identify a range of evidence for inclusion in the review and is unclear at this stage whether quality assessment will be possible, and which type of quality assessment tool will be most appropriate.

Revised QA: A suitable tool developed by Heirene et al. has been identified which could be used to assess the quality of the studies that report on the accuracy, validity and/or reliability of a cognitive assessment tool.

Analysis & synthesis

If feasible the final stage of the review will explore the differences in clinical criteria across the included studies and consider the factors that might explain where and why these differences occur, and the sources of clinical heterogeneity in diagnoses of ARBI. Studies will be grouped and organised to identify commonly applied criteria and patterns in diagnostic criteria within and across groups.

Example search strategy

Ovid MEDLINE(R) 1946 to October Week 1 2019

#	**Search term**
1	Alcohol-related disorders/ OR Alcoholism/ OR Ethanol/to
2	(alcoholi* OR (alcohol adj2 (dependen* OR excess* OR chronic OR "use disorder*" OR misuse OR addict*))).tw.
3	1 OR 2
4	((cognitive OR neurological OR neuropsychological OR neurocognitive OR neuropsychiatric) adj2 (impair* OR deficit* or disorder* or dysfunction* OR defect*)).tw.
5	(Korsakoff* OR "Korsakoff's syndrome" OR "Wernicke-Korsakoff" OR "Wernicke-Korsakow").tw.
6	Korsakoff Syndrome/ OR Cognitive Dysfunction/ OR Cognition Disorders/ OR Neurocognitive Disorders/ OR Brain Diseases/ OR "Brain Damage, Chronic"/ OR Brain Injuries/ OR Brain/pa OR exp Brain Diseases/ci
7	or/4-6
8	(alcohol* adj2 (amnestic OR dementia)).tw.
9	(alcohol* adj2 (brain OR cognitive) adj (injur* OR damage OR impair*)).tw.
10	Alcohol Amnestic Disorder/
11	Alcoholic Korsakoff Syndrome/
12	"Alcohol-Induced Disorders, Nervous System"/
13	or/8-12
14	(3 AND 7) OR 13

15	(screen* OR diagnos* OR assess* OR differentiat* OR discriminat* OR determinin* OR distinguish* OR confirmat* OR ascertain* OR detect* OR characteris* OR characteriz* OR identif* OR definition OR define*).tw.
16	exp Diagnosis/
17	15 OR 16
18	(tool* OR score* OR criteri* OR classification OR classify OR "clinical feature*" OR "clinical presentation*" OR "diagnostic feature*" or validat* OR reliability OR accura* OR specificity OR sensitivity OR reproducibility OR (predictive adj value) OR algorithm* OR prevalence OR significance OR DSM* OR ICD*).tw.
19	exp Diagnosis/cl, st
20	exp Validation Studies/
21	exp "Sensitivity and Specificity"/
22	exp Algorithms/
23	exp Neuropsychological Tests/
24	"Diagnostic and Statistical Manual of Mental Disorders"/ OR "International Classification of Diseases"/
25	exp Brain Disorder/cl OR exp Neurocognitive Disorders/cl
26	"Terminology as topic"/
27	or/18-26
28	14 AND 17 AND 27
