# Supplementary material for: Informing the development of diagnostic criteria for differential diagnosis of alcohol-related cognitive impairment (ARCI) among heavy drinkers: A systematic scoping review
Source: PLoS One. 2023 Feb 8;18(2):e0280749. doi: 10.1371/journal.pone.0280749 (PMC9907814; doi:10.1371/journal.pone.0280749)
Supplement: S1 Table — (PDF) [file pone.0280749.s002.pdf]

S2 Table. Search strategy example for Ovid MEDLINE(R)

#	**Search terms**
1	((Korsakoff* or Wernicke*) and alcohol*).ti,ab.
2	(Alcohol* adj2 (brain or cognitive or memory or neurological OR neuropsychological OR neurocognitive OR neuropsychiatric) adj (disorder* or deficit* or deficiency or impair* or damage* or injur* or dysfunction* or defect*)).ti,ab.
3	(Alcohol* adj2 (dementia or amnesic or amnestic)).ti,ab.
4	Alcoholic Korsakoff Syndrome/
5	Or/1-4
6	Alcoholism/ or Alcohol-Related Disorders/
7	Alcohol Amnestic Disorder/ or Brain Damage, Chronic/ or Korsakoff Syndrome/ or Dementia/ or Wernicke Encephalopathy/
8	6 and 7
9	5 or 8
