# Supplementary material for: Informing the development of diagnostic criteria for differential diagnosis of alcohol-related cognitive impairment (ARCI) among heavy drinkers: A systematic scoping review
Source: PLoS One. 2023 Feb 8;18(2):e0280749. doi: 10.1371/journal.pone.0280749 (PMC9907814; doi:10.1371/journal.pone.0280749)
Supplement: S2 Table — (PDF) [file pone.0280749.s003.pdf]

S4 Table. Critical appraisal of studies about the accuracy, validity and/or reliability of a cognitive assessment tool

Critical appraisal question	Study						
	**Brown et al., 2019**	**Wester et al., 2013**	**Errico et al., 1990**	**Ewert et al., 2018**	**Pelletier et al., 2018**	**Ritz et al., 2015**	**Jurado-Barba et al., 2017**
Was a sufficient period of abstinence (i.e., preferably >2 months but a minimum of >6 weeks) achieved prior to assessment?	Min 5 weeks	At intake	NIS 7 days after admission to treatment programme; NP tests 3 weeks after admission	Within 2 weeks and at least 7 days after alcohol withdrawal	7-10 days after alcohol withdrawal	Immediately after withdrawal	Not reported
Are participants diagnosed in accordance with (or already diagnosed using) the criteria outlined in nosological systems (DSM or ICD) or other accepted diagnostic criteria (e.g., Oslin & Cary, 2003)?	'In line' with Wilson for ARDB. KS based on ICD-10 Amnesic Syndrome.	'Suspected' cognitive impairment. AUD based on DSM-IV-TR.	Met National Council on Alcoholism criteria	Severe AUD assessed by DSM-V criteria	AUD based on DSM-IV	AUD based on DSM-IV	AUD based on DSM-V
Is an ARCI/AUD diagnosis confirmed using an appropriate reference standard* prior to or following neuropsychological assessment?	No	Unclear, extensive NP assessment undertaken as part of admission but not reported on.	Yes	Yes	Yes	Yes	Yes
Were persons with confounding conditions such as traumatic brain injuries or dementia excluded?	Severe brain injury excluded. Dementia not mentioned.	Not reported	No/Not clear	Yes. Exclusions were: severe comorbid neurological or psychiatric disease such as dementia; Alzheimer's disease; psychosis; past history of stroke or coma; encephalopathy and refusal to participate in the study; history of cardiac disease; infection by HIV.	Exclusion criteria were severe co-morbid neurological or psychiatric diseases such as dementia, Alzheimer's disease, psychosis, past history of stroke, coma, or encephalopathy, current consumption of cocaine and/or cannabis and/or heroin before admission, and refusal.	None of them had severe, enduring and global amnesia defining the Korsakoff's syndrome nor alcoholic dementia, Marchiafava-Bignami or central pontine myelinolysi	Yes. Exclusion criteria for both groups were presenting history of traumatic brain injury and unrelated to alcohol sudden brain injury or other neurological diseases; psychiatric comorbidity; being underage (<18 years old); and presenting consumption, abuse or dependence towards other substances.

Critical appraisal question	Study						
	**Brown et al., 2019**	**Wester et al., 2013**	**Errico et al., 1990**	**Ewert et al., 2018**	**Pelletier et al., 2018**	**Ritz et al., 2015**	**Jurado-Barba et al., 2017**
Are the neuropsychological tools used specific to the language and culture of the population tested?	Yes	Yes	Yes	Yes	Yes	Yes	Yes
Are administrators blind to diagnoses where the ARCI/AUD diagnosis has been established pre-assessment?	No	Not clear	Not clear	NP test admin blind to MoCA result	NP test admin blind to MoCA and BEARNI test results	Not clear	Not clear
Were participants matched according to demographic variables (age and gender) or were differences accounted for in between group comparisons?	Differences accounted for.	Yes	No/Not clear	N/A	N/A	No/Not clear	Not reported
Were participants matched according pre-morbid IQ or were differences accounted for in between group comparisons?	No. Occupational status.	No	No/Not clear	N/A	N/A	No/Not clear	Not reported
Were effect sizes reported to support analyses where appropriate?	Yes	No	No	N/A	Yes	No	No
Type 1 error potential: did the authors report adjusting the alpha level or consider the risk of type 1 error when multiple statistical comparisons made?	Yes. Bonferroni correction applied.	Bonferroni-corrected post-hoc tests	Not reported	Yes	Not reported	Yes. Bonferroni correction applied.	Not reported
