# Supplementary material for: Informing the development of diagnostic criteria for differential diagnosis of alcohol-related cognitive impairment (ARCI) among heavy drinkers: A systematic scoping review
Source: PLoS One. 2023 Feb 8;18(2):e0280749. doi: 10.1371/journal.pone.0280749 (PMC9907814; doi:10.1371/journal.pone.0280749)
Supplement: S1 Appendix — (PDF) [file pone.0280749.s004.pdf]

S3 Appendix. Complete data extraction tables

S3A Table. Complete data extraction tables for ARBI patient samples.

Author, Year	Methods		Outcomes			
	Patient characteristics	Procedures and Analysis	Between group comparisons	Validity	Sensitivity (Se) & Specificity (Sp)	Other
Brown et al., 2019 (19)	**Aim:** To evaluate the suitability of ACE-III and RBANS for the assessment and screening of ARBI<br>**Eligibility criteria:** Diagnosis of KS or ARBI based on Wilson 2012 criteria. Origin of diagnoses varied, and exact procedures used to make diagnostic decisions unknown.<br>**Recruitment from/Setting:** Glasgow specialist ARBD service and others (for AUD)<br><br>**Sample**<br>**No. patients:** 28 ARBI (11 KS, 17 other)<br>**Comparator:** 31 AUD<br>**Exclusions:** Severe brain injury, serious physical or psychological complications.<br>**Gender, % male:** AL, 63%; ARBD, 64%<br>**Mean age (SD):** AL, 46.1 y (8.9); ARBD 56.9 y (7.2)<br>**Level of education:** NR<br>**Alcohol use history (median y drinking):** AL, 16 y (range 3-55); ARBD, 20 y (range 2-40)	**Assessment(s):** ACE-III (tests five cognitive domains: attention, memory, verbal fluency, language, and visuospatial function); RBANS (12 subtests which provide five index scores: Immediate memory, Visuospatial/ constructional, Language, Attention, and Delayed memory. Combining these index scores provides an overall performance score).<br>**NP battery:** NA<br>**Period of abstinence:** Abstinent from alcohol and other substances for min. 5 weeks.<br>**Analysis:** Shapiro-Wilk's test and Welch's t-test for between-group comparisons. ROC analyses to determine screening accuracy.	*ACE-III:* ARBI patients scored sig. lower than AUD on total scores (Cohen's *d* = 1.14), attention (0.94), memory (1.20) and fluency (0.92).<br>*RBANS:* ARBI patients scored sig. lower than AUD on total scores (1.46), immediate memory (1.47), and delayed memory (1.70).	Both groups' total ACE-III and RBANS scores were significantly and strongly correlated (AUD, r = 0.784, p<0.001; ARBI, r = 0.700 p<0.001).	Total scores on both tests distinguished between ARBI and AUD patients.<br>*ACE-III:* AUC = 0.823, 95%CI: 0.71-0.93, p < 0.001); Optimal cut-off: ≤86 (Se 0.82, Sp 0.73)<br>*RBANS:* AUC 8.46, 95%CI: 0.75-0.95, p < 0.001); Optimal cut-off ≤83 (Se 0.89, Sp 0.67)	…

	**Global cognitive function:** NR					
Carlen et al., 1994 (20)	**Aim:** To examine the distribution of ARD in a sample of long-term care facility residents<br>**Eligibility criteria:** Residents in five long-term care facilities in Canada; aged 50 or over.<br>**Recruitment from/Setting:** Long-term care facilities in Canada; psychiatric hospital, chronic care ward of a general hospital and two nursing homes.<br><br>**Sample**<br>**No. patients:** 130 patients (46 AD; 25 VaD; 31 ARD; 28 misc. dementias)<br>**Comparator:** NA<br>**Exclusions:** Mental retardation, profound deafness, active schizophrenia, or selective language impairment.<br>**Gender, % male:** AD, 30%; VaD, 52%; ARD, 74%; misc., 46%<br>**Mean age (SD):** AD, 84 y (7.8); VaD, 82 y (8.2); ARD, 73 y (10.6); misc., 80 y (9.4)<br>**Level of education:** NR<br>**Alcohol use history:** NR<br>**Global cognitive function (mean CDR score):** AD, 3.5 (SD 1.5); VaD, 2.3 (SD 1.5); ARD, 1.6 (SD 1.1); misc., 1.5 (SD 1.4)	**Assessment(s):** Participants were screened for cognitive impairment with the CERAD NP battery and the MMSE. Diagnosis of ARD based on an indication of a history of prolonged (>5 years just before admission) and excessive alcohol use (>6 oz/day on average), a lack of progression in cognitive decline (for at least the 1st year subsequent to institutionalization), and exclusion of other causes of dementia.<br>**NP battery:** NA<br>**Period of abstinence:** NA<br>**Analysis:** Not clear, chi-square for between group comparisons.	Patients in the ARD group were younger and had been institutionalised for longer (mean length of stay 7.5 y (SD 5.4) vs. AD 4.2 y (SD 3.1), VaD 4.0 y (SD 4.2), misc. 4.3 y (SD 2.7).	…	…	24% of participants had a diagnosis of ARD

Gilchrist & Morrison 2005 (21)	**Aim:** To describe the prevalence of ARBD among hostel residents in Glasgow<br>**Sample/Eligibility criteria:** Homeless people aged >34 years from five large hostels<br>**Recruitment from/Setting:** Homeless hostels in Glasgow<br><br>**Sample**<br>**No. patients:** 266<br>**Comparator:** NA<br>**Exclusions:** None<br>**Gender, % male:** 89%<br>**Mean age (SD):** 53 y (NR)<br>**Level of education:** NR<br>**Alcohol use history (met criteria for lifetime dependence):** 61%<br>**Global cognitive function:** see Outcomes	**Assessment(s):** Participants were screened for cognitive impairment and alcohol dependence: ACE (score <88 indicating cognitive impairment), FAST, and LDQ (if FAST; last week ≥9) or CAGE (≥2). Psychiatrist and psychologist clinically assessed respondents who met criteria for lifetime AUD and CI.<br>**NP battery:** NA<br>**Period of abstinence:** NA<br>**Analysis:** Applied age-specific prevalence rates.	…	…	…	82% had a score <88 on ACE.<br><br>Estimated that 21% (CI 16-26%) of the homeless hostel dwellers had ARBI.
Horton et al., 2015 (32)	**Aim:** Investigate the neurocognitive, psychosocial and everyday functioning of a group of individuals with ARBD<br>**Eligibility criteria: '**Diagnosed with ARBD' and admitted to facility in previous 3 weeks.<br>**Recruitment from/Setting:** Abstinence-based 'ARBD residential rehabilitation service'	**Assessment(s):** Neurocognitive assessment with National Adult Reading Test and CANTAB. Abstinent for at least 3 months. Standardised self-report to assess quality of life, mental health status and mood disturbance: EQ-5D-5L, SF-36v2, POMS, HADS, DASS. Functional assessment with AMPS.<br>**NP battery:** NA	Group *Z* scores showed participants performed below the normative average on all 5 subtests. % in impaired Z score category: PAL (episodic memory), 50–65%; RVP	…	…	…

	**Sample**<br>**No. patients:** 20 ARBI<br>**Comparator:** No controls. CANTAB scores compared to normative reference database.<br>**Exclusions:** None<br>**Gender, % male:** 75%<br>**Mean age (SD):** 53 y (NR)<br>**Level of education:** NR<br>**Alcohol use history:** NR<br>**Global cognitive function:** see Outcomes	**Period of abstinence:** NA<br>**Analysis:** Descriptive statistics calculated for each measure. CANTAB performance on 5 tasks (PAL, RVP, SOC, SWM and IED) compared to a normative reference database of adults using standardised *Z* scores. Z scores >2 SD below the normative mean (Z=>-2) outside normal range (i.e. impaired).	(sustained visual attention), 30-60%; SOC (spatial planning), 5-50%; SWM (working memory), 5%; IED (rule acquisition and attentional set shifting), 20%.			
Oslin & Cary 2003 (22)	**Aim:** To validate the diagnostic criteria for ARD<br>**Sample/Eligibility criteria:** All consecutively admitted residents to a nursing home. 158 given a clinical diagnosis of 'probable' dementia: ARD, AD or VaD (according to NINCDS/ADRDA criteria and California criteria for VaD). Oslin 1998 diagnostic criteria for probable ARD.<br>**Recruitment from/Setting:** Veteran Affairs Nursing Home Care Unit<br><br>**Sample**<br>**No. patients:** 158 patients (16 'probable' ARD; 26 AD; 46 VaD; 70 mixed or unknown origin)<br>**Comparator:** NA	**Assessment(s):** Clinical assessment at baseline and 2-year follow-up, assessed cognitive and affective status (MMSE) as well as the presence of psychotic symptoms and a history of alcohol abuse (CLDQ).<br>**NP battery:** NA<br>**Period of abstinence:** NA<br>**Analysis:** Mann-Whitney or Kruskal-Wallis and chi-square for between group comparisons. Random-effects linear model for change over time.	Comparison of mean changes between ARD and AD suggests sig. Time x Group interaction (controlled for b/s MMSE and age).	…	…	…

	**Exclusions:** Tests not conducted within 2 months of admission, 'too ill', refused, aphasic and died/discharged before interview.<br>**Gender, % male:** ARD, 100%; AD, 96%; VaD, 100%; mixed, 100%<br>**Mean age (SD):** ARD, 66.8 y (11.7); AD, 74.7 y (9.7); VaD, 74.3 y (9.2); mixed, 73.2 y (10.4)<br>**Level of education:** NR<br>**Alcohol use history (lifetime abuse/dependence):** ARD, 100%; AD, 28.0%; VaD, 43.5%; mixed, 44.8%<br>**Global cognitive function (mean MMSE score):** ARD, 20.8 (6.9); AD,14.4 (10.2); VaD, 13.9 (8.8); mixed, 13.7 (8.8)					
Schmidt 2005 (23)	**Aim:** To compare the neuropsychological profile of ARD with those of typical cortical and subcortical dementia<br>**Sample/Eligibility criteria:** Based on Oslin et al., 1998: (1) 35 alcoholic drinks per week for men (28 for women) for >5-year period, (2) a diagnosis of dementia at least 60 days after last exposure to alcohol, (3) no history of an acute onset of symptoms associated with Wernicke's encephalopathy (e.g. global confusional state, ocular abnormalities), and	**Assessment(s):** Comprehensive neuropsychological protocol that included tests of executive control, language, and memory was administered. Executive function (Clock Drawing, Boston Revision of the WMS Mental Control subtest); Language (WAIS-R Similarities subtest, Boston Naming Test); Memory - learning, delayed recall and recognition (PrVLT).<br>**NP battery:** As above	ANOVA showed sig. differences in performance across groups. No sig. differences between ARD and VaD on executive control, language, or memory. ARD vs. AD had sig. lower scores on executive control	…	…	…

	(4) a lack of focal neurological signs (except ataxia or peripheral sensory polyneuropathy).<br>**Recruitment from/Setting:** Participating in a memory assessment programme<br><br>**Sample**<br>**No. patients:** 14 ARD<br>**Comparator:** 15 AD, 13 VaD and 20 controls. All dementia diagnoses based on multidisciplinary team assessment.<br>**Exclusions:** NR<br>**Gender, % male:** ARD, 50%; AD, 27%; VaD, 23%; mixed, 30%<br>**Mean age (SD):** ARD, 80.3 y (6.1); AD, 77.7 y (5.4); VaD, 79.9 y (5.3); 75.9 y (9.5)<br>**Level of education:** ARD, 11.6 y (SD 2.6); AD, 12.3 y (SD 2.5); VaD, 12.2 y (SD 3.0); mixed, 14.0 y (SD 2.7).<br>**Alcohol use history:** NR<br>**Global cognitive function (mean MMSE score):** 22.4 (SD 3.3); 23.2 (SD 1.9); 22.9 (SD 2.4); 28.6 (SD 1.0)	**Period of abstinence:** NA<br>**Analysis:** Calculated Z-scores and ANOVA used to compare across groups. Post-hoc Tukey tests to further describe differences between groups. Groups matched for dementia severity.	(Mental Control, p=0.020; Clock Drawing, p=0.025). ARD vs. AD had sig. higher scores on delayed recognition memory task (p=0.004).			
Thompson 2020 (7)	**Aim:** To explore 12-month period prevalence of ARBI in AUD patients<br>**Sample/Eligibility criteria:** All inpatients aged 18 years and over who were reviewed by the Alcohol Care Team's Specialist	**Assessment(s):** Patients matching at least one of the following was considered high risk and was assessed for evidence of ARBI using the MoCA tool: 1) more than three alcohol-related admissions in	…	…	…	12-month period prevalence of at least 1 high-risk criteria: 16.1% (n=205). Prevalence of

	Nurses between April 1, 2017 and March 31, 2018<br>**Recruitment from/Setting:** Acute hospital, UK<br><br>**Sample**<br>**No. patients:** 1,276<br>**Comparator:** NA<br>**Exclusions:** NR<br>**Gender, % male:** 69%<br>**Mean age (SD):** 52 y (14)<br>**Level of education:** NR<br>**Alcohol use history (mean AUDIT score):** 19.3 (SD 9.9)<br>**Global cognitive function:** see Outcomes	one year; 2) two alcohol-related admissions in any given 30-day period; and 3) patient or their significant other had concerns regarding cognition.<br>**NP battery:** NA<br>**Period of abstinence:** NA<br>**Analysis:** Period prevalence rate of MoCA ≤23 estimated (≤21 and ≤25 for sensitivity analyses). Formal analysis undertaken using prevalence rate ratios, presented with 95% confidence intervals.				MoCA score in high-risk cohort: ≤23, 36.1%; ≤21, 26.3%; ≤25, 53.7%.<br><br>No sig. difference on any of the investigated variables (gender, age, within-study deprivation, smoker, AUDIT).
Wester 2013 (24)	**Aim:** To examine the discriminatory power of the MoCA and predictive capacities for the severity of amnesia<br>**Eligibility criteria:** Suspected cognitive impairments due to alcohol-use disorder. KS (DSM-IV-TR criteria or alcohol-induced persisting amnestic disorder), non-KS forms of ARBI and healthy controls.<br>**Recruitment from/Setting:** Korsakoff Clinic, Vincent van Gogh Institute for Psychiatry<br><br>**Sample**	**Assessment(s):** Dutch version of MoCA and the RBMT-3. RBMT-3 used to categorise participants based on memory impairments: severe, GMI <70; mild, GMI 70-84; unimpaired, GMI ≥85.<br>**NP battery:** NA<br>**Period of abstinence:** Unclear, MoCA administered at intake and RBMT-3 approx. 6-8 weeks after admission.<br>**Analysis:** MANCOVA to compare MoCA total score, domain scores	Total scores, and memory subdomain significantly differed by group (both p<0.001)	…	Total score distinguished: ARBI vs. controls (AUC 0.85, p <0.001); optimal cut-off ≤24 (Se 0.85, Sp 0.69)<br>Unimpaired vs. severe (AUC 0.96, p<0.001); optimal cut-off ≤23 (Se 0.91, Sp 0.88)	…

	**No. patients:** 26 ARBI (non-KS)<br>**Comparator:** 20 KS; 33 controls<br>**Exclusions:** Not reported<br>**Gender, % male:** ARBI, 77%; KS, 75%; controls, 46%<br>**Mean age (SD):** ARBI, 54.5 y (8.1); KS, 57.6 y (8.7); controls, 53.0 y (6.7)<br>**Level of education:** ARBI, 4 (range 1-6); KS, 3 (range 2-6); controls, 5 (range 3-6)<br>**Alcohol use history:** NR<br>**Global cognitive function (mean MoCA score):** ARBI, 22.0 (SD 3.8); KS, 18.9 (SD 3.7); controls, 26.5 (SD 2.0)	with the RBMT-3 GMI score. ROC analyses to examine whether MoCA differentiates between controls, ARBI (non-KS) and KS patients and for optimal cut-off scores (at Se ≥0.80 and Sp ≥0.60).			Unimpaired vs. mild (AUC 0.82, p<0.001); optimal cut-off ≤24 (Se 0.88, Sp 0.71)<br>No optimal cut-off score to distinguish ARBI (non-KS) from KS.	
Wilson 2012 (11)	**Aim:** To describe the clinical presentation, course and psychosocial outcome of patients with ARBD<br>**Sample/Eligibility criteria:** Patients referred into a tertiary service designed to cater for patients with severe ARBI.<br>**Recruitment from/Setting:** Tertiary service designed to cater for patients with severe ARBI. Screened according to adapted criteria from Oslin & Cary 2003.<br><br>**Sample**<br>**No. patients:** 41<br>**Comparator:** NA<br>**Exclusions:** NR	**Assessment(s):** ACE-R and HoNOS-ABI (measure of neuropsychiatric sequelae following brain injury) assessments.<br>**NP battery:** NA<br>**Period of abstinence:** Unclear, abstinence strongly recommended for those attending the service<br>**Analysis:** Descriptive statistics.	…	…	…	Group average ACE-R score (n=22): 65.7 (range 30-93). HoNOS-ABI (n=26): 10 rated as experiencing severe cognitive problems rest had circumscribed cognitive deficits or milder impairment. 23 experienced confabulations,

	**Gender, % male:** 73%<br>**Mean age (SD):** 54 y (range 43-68)<br>**Level of education:** NR<br>**Alcohol use history:** NR<br>**Global cognitive function:** see Outcomes					hallucinations or delusions.

S3B Table. Complete data extraction tables for AUD patient samples.

Author, Year	Methods		Outcomes			
	**Patient characteristics**	**Procedures and Analysis**	**Between group comparisons**	**Validity**	**Sensitivity (Se) & Specificity (Sp)**	**Other**
Alarcon 2015 (30)	**Aim:** To explore the potential utility of MoCA<br>**Sample/Eligibility criteria:** Dependence on alcohol based on DSM-IV<br>**Recruitment from/Setting:** Addiction Treatment Unit, Teaching Hospital<br><br>**Sample**<br>**No. patients:** 166 AUD<br>**Comparator:** NA. Participants categorised into 3 groups based on MoCA score.<br>**Exclusions:** Severe comorbid neurologic or psychiatric disease (e.g. dementia, AD, psychosis, past history of stroke or coma, encephalopathy)<br>**Gender, % male:** 63%<br>**Mean age (SD):** 49.9 (9.2)	**Assessment(s):** Used version 7.1 MoCA (explores 8 cognitive domains). Participants abstinent for 1-2 weeks.<br>**NP battery:** NA<br>**Period of abstinence:** 1-2 weeks<br>**Analysis:** Comparisons with Student's *t*-test or nonparametric Wilcoxon test, or chi-square test or Fisher's exact test. Multivariate analysis performed using binary logistic regression.	68.1% scored ≤26. Categorised into 3 groups with low (≥26, n=46), medium (22 to 25, n=65) and high scores (≤21, n=42).<br><br>Age and sex were not related to scores. Having a high education level (>12 years) significantly increased the likelihood of a high score (RR 3.26,	…	…	…

	**Level of education:** <12 y, 67.3%; 12 y, 18.9%; >12 y, 13.8%<br>**Alcohol use history (mean duration):** 14.2 y (SD 8.6)<br>**Global cognitive function (mean MoCa score):** 23.5 (SD 3.5)		95%CI 1.25-8.47, p=0.01).<br><br>Sig. difference (p<0.05) in subscores across 3 groups: Low vs. medium = visuospatial, attention-subtraction, language-fluency, abstraction, delayed recall, orientation.<br>Low vs. high = visuospatial, attention-subtraction, language-fluency, abstraction, delayed recall, orientation.<br>Medium vs. high = visuospatial, attention-list of digits, language-fluency,			

			abstraction, delayed recall			
Cao et al., 2021 (31)	**Aim:** To identify whether the RBANS scale and ERPs could be verified with each other and provide an objective basis for early identification and diagnosis of cognitive impairment of patients with AUD.<br>**Eligibility criteria:** Alcohol-dependent patients, met DSM-IV-TR; education level junior high school or greater; aged 18–60 years; Han nationality; completed acute withdrawal treatment for 7 days with no withdrawal symptoms.<br>**Recruitment from/Setting:** Psychiatry Department of the Second People's Hospital of Hunan Province<br><br>**Sample**<br>**No. patients:** 60 male AUD<br>**Comparator:** 40 male controls<br>**Exclusions:** (1) having past and current history of brain injury, cerebral mental illness or other mental disorders; (2) substances other than nicotine dependence prior to entering the group; (3) consciousness disorders and delirium; (4) severe heart, liver, and kidney dysfunction; (5) pregnant and lactating women.	**Assessment(s):** RBANS (1998 version; digital breadth, coding test, picture naming, word fluency test, graphic reproduction, line positioning, vocabulary learning, story learning, vocabulary recall, vocabulary re-recognition, story recall, and graphical recall). Event-related potentials (ERPs) assays; analysis index was the incubation period and the amplitude of P300 and P200, and the incubation period of N100 and N200 at Cz point.<br>**NP battery:** NA<br>**Period of abstinence:** 7 days<br>**Analysis:** Comparisons with Student's *t*-test; correlations analysed by partial correlation analysis. Adjusted with Bonferroni correction.	Original scores of speech function (P=0.004), attention function (P=0.001), delayed memory (P=0.000), and immediate attention (P=0.000) in the alcohol-dependent group were significantly reduced compared to controls. No difference on visual breadth (P=0.054).			

	**Gender, % male:** 100%<br>**Mean age (SD):** AUD, 42.33 y (SD 7.57); controls, 42.03 y (SD 6.61)<br>**Level of education:** AUD, 9.00 y (SD 1.85); controls, 9.58 y (SD 2.11)<br>**Alcohol use history (mean y alcohol dependence):** 11.30 y (6.94)<br>**Global cognitive function:** Not reported					
Errico et al., 1990 (25)	**Aim:** To assess whether the NIS could serve as a valid indicator of NP impairment in patients with AUD<br>**Eligibility criteria:** Men with alcohol dependence (AUD; National Council on Alcoholism criteria); 24-60 years; at least 6th grade education.<br>**Recruitment from/Setting:** Oklahoma City Veterans Administration Medical Center<br><br>**Sample**<br>**No. patients:** 73 AUD<br>**Comparator:** 254 controls<br>**Exclusions:** Not reported<br>**Gender, % male:** 100%<br>**Mean age (SD):** AUD, 42.5 y (SD 9.7); controls, 43.0 y (SD 8.5)<br>**Level of education:** NR	**Assessment(s):** NIS, participants rate the intensity with which they experience symptoms on a 4-point scale: GMI (total raw-score sum of 45 neuropsychological item); TIC (global indicator of impairment; SIM (derived by dividing GMI by TIC). LIE scale (five items that assess test-taking attitude); GEN (questions about difficulties in mental efficiency, alertness and endurance); PAT (items that frequently suggest the presence or history of neuropsychological impairment); LV (reflects complaints of verbal communication and learning problems); and FRU (items reflecting undue affective and motivational reactions).	AUD patients scored higher than controls on NIS (p<0.0001). ALC and controls differed on all scales except the LIE scale.	Five NIS scales (PAT, GMI, LV, SIM and TIC) correlated with most NP test clusters. GEN scale significantly correlated with learning/memory and performance index. LIE and FRU scales not significantly correlated with task performance.	…	

	**Alcohol use history (mean y problem drinking):** AUD, 11.5 y (SD NR); controls, NA<br>**Global cognitive function (mean score on NIS-PAT scale):** AUD, 5.1 (SD 5.4); controls, 1.3 (SD 2.1)	**NP battery:** WMS, Symbol-Digit Paired Associates, Luria Memory Words, Face-Name Paired Associates, Verbal Paired Associates, WAIS-R Block Design, WAIS-R Digit Symbol, Twenty Questions, Abstraction Test, Hypothesis Testing Procedure, Adaptive Skills Battery, Conceptual Level Analogy Test, RRB [Lafeyette Pegboard, Sentence Writing, TMT-B])<br>**Period of abstinence:** NIS, 7 days after admission to treatment programme; NP battery, 3 weeks after admission<br>**Analysis:** MANOVA for comparison between AUD patients and controls. Pearson correlation between each of the NIS scales and three clusters of NP tests (learning and memory, problem solving, perceptual-motor skills). Overall performance index constructed from mean level of performance on all tests.				

Ewert et al., 2018 (26)	**Aim:** To compare MoCA scores in patients hospitalized for AUD with and without cognitive impairment<br>**Eligibility criteria:** Admission for 'severe' AUD assessed by DSM-V criteria; age above 18 years; ability to understand and speak French; oral agreement to participate.<br>**Recruitment from/Setting:** Hospital-based substance use disorder rehabilitation centre<br><br>**Sample**<br>**No. patients:** 56 AUD (CI, 31; NCI, 25)<br>**Comparator:** NA<br>**Exclusions:** Severe comorbid neurological or psychiatric disease such as dementia; AD; psychosis; past history of stroke/coma; encephalopathy; refusal to participate; past or present drug consumption except tobacco; history of cardiac disease; HIV.<br>**Gender, % male:** NR<br>**Mean age (SD):** NCI, 49.3 y (7.9); CI, 49.6 y (9.4)<br>**Level of education (≤ 12 y):** NCI, 60%; CI, 90%<br>**Alcohol use history (mean quantity):** NCI, 200 g/day (SD 90); CI, 214 g/day (SD 120)<br>**Global cognitive function (mean MoCa score):** NCI, corrected 27.6 (SD 1.8) /	**Assessment(s):** MoCA systematically administered on admission; complete NP evaluation performed when the MoCA score falls below the usual normal value (≤26) or on special request of the physician but not routinely done when the MoCA test is normal. For this study, all admitted patients fulfilling the inclusion criteria listed above underwent an extensive NP evaluation regardless of MoCA score (within 2 weeks of admission and at least 7 days after alcohol withdrawal).<br>**NP battery:** CVLT, WAIS digit span, TMT, Stroop test, ROCF<br>**Period of abstinence:** Within 2 weeks and at least 7 days after alcohol withdrawal<br>**Analysis:** Pearson correlation analysis between global score and NP tests. Omnibus *F*-statistic used to check accuracy of classification. ROC analyses to determine cut-off scores.	…	Total score correlated significantly with NP tests.	Uncorrected score: AUC 0.87 (95%CI: 0.77-0.96); cut-off ≤26.<br>Corrected score: AUC 0.84 (95%CI: 0.73-0.95); cut-off ≤27.	

	uncorrected 27.0 (SD 1.9); CI, corrected 24.0 (SD 3.3) / uncorrected 23.1 (SD 3.3)					
Jurado-Barba et al., 2017 (27)	**Aim:** To assess the main neurocognitive deficits in patients with AUD<br>**Eligibility criteria:** AUD patients (DSM-V) attending for psychotherapy<br>**Recruitment from/Setting:** Hospital's Addictive Behaviour Unit (Madrid)<br><br>**Sample**<br>**No. patients:** Phase 2, 90 AUD<br>**Comparator:** Phase 2, 88 controls<br>**Exclusions:** Presenting history of TBI and unrelated to alcohol sudden brain injury or other neurological diseases; psychiatric comorbidity; aged <18 years old; presenting consumption, abuse or dependence towards other substances.<br>**Gender, % male:** AUD, 67%; controls, 56%<br>**Mean age (SD):** 48 y (10.3)<br>**Level of education (≤ high grade):** AUD, 88%; controls, 44%<br>**Alcohol use history (mean y alcohol dependence):** AUD, 16 (SD 10.4); controls, NA<br>**Global cognitive function:** NR	**Assessment(s):** TEDCA. Items composing the test were chosen at the end of phase 1 from NP tests.<br>**NP battery:** ROCF, BVMGT, Texts I and II, Direct and Inverse Digits from WMS-III, TMT-A and -B, Similarities Test, Matrices Test and Digit Symbol Coding test from WAIS-IV, the semantic and phonological verbal fluency test (F-A-S test), a Go-No Go task and the Zoo test (BADS).<br>**Period of abstinence:** Not reported.<br>Difference between groups analysed with Students' *t*-test. Cronbach's alpha coefficient for reliability and internal consistency analysis.<br>**Analysis:** ANOVA for discrimination capacity. ROC analysis for diagnostic validity.	AUD patients scored significantly lower than controls.	…	AUC 0.80 (95%CI 0.74-0.86); optimal cut-off: ≤10.5 (Se 0.67, Sp 'close to' 0.77)	

Pelletier et al., 2018 (28)	**Aim:** To compare the performance of MoCA and BEARNI<br>**Eligibility criteria:** Patients admitted for AUD; dependence on alcohol assessed by DSM-IV.<br>**Recruitment from/Setting:** Rehabilitation centre<br><br>**Sample**<br>**No. patients:** 90 AUD<br>**Comparator:** NA<br>**Exclusions:** Severe co-morbid neurological or psychiatric diseases such as dementia; AD; psychosis; past history of stroke, coma, or encephalopathy; current consumption of cocaine, cannabis and/or heroin before admission; refusal.<br>**Gender, % male:** 74%<br>**Mean age (SD):** 48.9 y (9.6)<br>**Level of education (≤12 y):** 79%<br>**Alcohol use history (duration of heavy drinking):** 15.6 y (SD 10.3)<br>**Global cognitive function ('cognitive trouble'):** none, 51%; mild/moderate, 31%; severe, 18%	**Assessment(s):** Used version 7.1 MoCA (explores 8 cognitive domains) and version of BEARNI proposed by Ritz et al 2015.<br>**NP battery:** TMT; the Stroop test; adapted GREFEX version and fluency tasks; FCSRT; digit span subtest of the WAIS; ROCF.<br>**Period of abstinence:** Tests administered about 7-10 days after alcohol withdrawal<br>**Analysis:** Mean scores compared using Students' *t*-test or non-parametric Wilcoxon test. ROC analyses to determine BEARNI cut-off scores.	…	Both tests matched well with the NP tests.	*MoCA:* Se 0.79; Sp 0.65; PPV 0.69; 'well-classified' 0.72<br>*BEARNI Total:* Se 1.00; Sp; 0.02 PPV 0.50; 'well-classified' 0.50.<br>Cut-off: ≤14 (Se 0.82; Sp 0.68)	
Rao 2016 (33)	**Aim:** To examine the assessment of cognitive impairment in community services for older people	**Assessment(s):** ACE-III; five cognitive domains (attention, memory, verbal fluency, language				76% below the cut-off point of 82 on ACE-III.

	**Sample/Eligibility criteria:** Alcohol use disorder identified as drinking above recommended daily or weekly limits (>14 UK units/wk, women; >21 units/wk, men), diagnosis of harmful alcohol use according to ICD 10, an ICD 10 diagnosis of alcohol dependence or clinical documentation of alcohol withdrawal syndrome. Screened using the Addenbrooke's Cognitive Assessment Third edition (ACE-III).<br>**Recruitment from/Setting:** People under the caseloads of 4 community mental health teams within South London and Maudsley NHS Foundation Trust<br><br>**Sample**<br>**No. patients**: 25<br>**Comparator:** NA<br>**Exclusions:** Not reported<br>**Gender, % male:** 60%<br>**Mean age (range):** Not reported (67-86 y)<br>**Level of education:** Not reported<br>**Alcohol use history:** Not reported<br>**Global cognitive function:** Not reported	and visuospatial abilities). Cut-off points derived from normative data for older people.<br>**NP battery:** NA<br>**Period of abstinence:** Not reported<br>**Analysis:** Chi-squared applied to categorical data; unpaired t-tests for continuous data.				Scored below pre-defined cut-off score:<br>Attention/orientation: 86%<br>Fluency: 91%<br>Visuospatial function: 68%<br>Language: 45%<br>Memory: 73%
Reid et al., 2002 (34)	**Aim:** To determine whether older cognitively impaired adults with AUD manifest distinctive cognitive profiles	**Assessment(s):** Cognitive function assessed with MMSE. Calculated average weekly alcohol exposure	MANOVA showed no difference in total scores across alcohol exposure	…	…	

	**Sample/Eligibility criteria:** Older adults aged ≥65 years who received comprehensive geriatric assessments<br>**Recruitment from/Setting:** Geriatric Assessment Centre, hospital based<br><br>**Sample**<br>**No. patients:** 801<br>**Comparator:** NA. Categorised into two groups based on MMSE score (<24, ≥24)<br>**Exclusions:** Did not receive cognitive assessment, too impaired to undergo tests, proxy not available, aged <65 years, unable to locate medical records.<br>**Gender, % male:** MMSE <24, 29%; ≥24, 29%<br>**Mean age (SD):** MMSE <24, 79.8 y (7.2); ≥24, 77.5 y (7.7)<br>**Level of education (mean):** MMSE <24, 10.8 y (3.7); ≥24, 12.9 y (2.8)<br>**Alcohol use history (history of use/dependence):** MMSE <24, 6.2%; ≥24, 8.5%<br>**Global cognitive function (mean MMSE score):** MMSE <24, 17.1 (SD 5.4); ≥24, 26.5 (SD 1.9)	and reviewed medical records to assess for presence of AUD.<br>**NP battery:** NA<br>**Period of abstinence:** Not reported<br>**Analysis:** Group comparisons with *t*-tests or chi-square. ANOVA used to assess differences in total MMSE scores. Assessed difference on MMSE in two groups based on <24 and ≥24.	categories or on history of alcohol abuse or dependence for patients with MMSE <24.			
Ritz et al., 2015 (29)	**Aim:** To assess the validity and psychometric properties of BEARNI	**Assessment(s):** BEARNI, total score and cognitive score	AUD patients performed more	…	*Cut-off scores*	

	**Eligibility criteria:** DSM-IV criteria for AUD. Early in abstinence.<br>**Recruitment from/Setting:** Receiving withdrawal treatment as inpatients at Caen University Hospital<br><br>**Sample**<br>**No. patients:** 73 AUD<br>**Comparator:** 254 controls; 58 underwent the NP battery<br>**Exclusions:** Severe, enduring and global amnesia defining KS, alcoholic dementia; Marchiafava-Bignami disease; CPM<br>**Gender, % male:** AUD, 73%; controls, 47%<br>**Mean age (SD):** AUD, 45.5 y (8.9); controls, 42.8 y (10.9)<br>**Level of education (mean):** AUD, 11.2 y (SD 1.7); controls, 42.8 y (SD 10.9)<br>**Alcohol use history (mean AUDIT score):** AUD, 29.9 (SD 6.3); controls, 2.8 (SD 1.7)<br>**Global cognitive function (mean MMSE score):** AUD, 27.9 (SD 1.9); controls, 28.8 (SD 1.1)	**NP battery:** CVLT; verbal and visuospatial spans of the WMS-III; Letter-Number Sequencing subtest of the WAIS-III; TMT; RFFT; the Stroop test, and fluency tasks; ROCF; Walk-a-Line Ataxia Battery<br>**Period of abstinence:** Immediately after withdrawal<br>**Analysis:** Exploratory factor analysis for validation of internal structure. MANOVA and Tukey's post-hoc tests for comparisons of AUD and controls. Standardisation of two total scores using data from 58 controls. ROC analyses to determine cut-off scores.	poorly than controls (effects of group, p<0.001). Effect also found for each sub-test, except delayed verbal memory.		Mild: ≤19 (Se 0.83, Sp 0.67);<br>Moderate-to-severe: ≤16 (Se 0.80, Sp 0.77)<br>*Psychometric properties*<br>Mild: AUC 0.92; Se 0.98, Sp 0.50; PPV 0.93<br>Moderate-to-severe: AUC 0.84; Se 0.95, Sp 0.71; PPV 0.79<br><br>Sub-test scores: Se all 1.0; Sp 0.752-0.996; AUC 0.950-0.998	
Taylor 1997 (35)	**Aim:** To assess the potential of the MST to detect cognitive impairment in patients with AUD undergoing detoxification.	**Assessment(s):** Administered the MST (consisted of 3 tasks; Word recall, Sentence recall and Figure recall) and AUDIT.	AUD patients made a greater number of errors on each recall task	…	…	

	**Sample/Eligibility criteria:** AUD (‘hazardous drinker’) based on AUDIT score.<br>**Recruitment from/Setting:** Detoxification unit<br><br>**Sample**<br>**No. patients:** 16 AUD; 3 had ARBI diagnosis (1 at a specialist unit, 2 based on CT scans)<br>**Comparator:** 16 controls.<br>**Exclusions:** Not reported<br>**Gender, % male:** AUD,<br>**Mean age (SD):** AUD, 36.8 y (7.1); controls, 37.8 y (7.3)<br>**Level of education (mean):** AUD, 10.9 y (SD 2.4); controls, 11.9 y (SD 1.5)<br>**Alcohol use history (mean AUDIT score):** AUD, 33.1 (SD 6.0); controls, 2.9 (SD 2.0)<br>**Global cognitive function:** NR	**NP battery:** NA<br>**Period of abstinence:** Not reported<br>**Analysis:** Group comparisons with Students’ *t*-test. Controls matched on age, gender and education.	than controls but no sig. difference in mean number of errors between groups on individual task. Group mean for total errors was sig. higher in AUD patients ($p<0.0133$).			

ACE, Addenbrooke’s Cognitive Examination; AD, Alzheimer’s Disease; ARBI, alcohol-related brain injury; AUC, Area Under the Curve; AUD, alcohol dependence; BADS, Behavioural Assessment of the Dysexecutive Syndrome; BEARNI, Brief Evaluation of Alcohol-Related Neuropsychological Impairments; BVMGT, Bender Visuo-Motor Gestaltic Test; CPM, Central Pontine Myelinolysis; CVLT, California Verbal Learning Test; FCSRT, Free and Cued Selective Reminding Test; KS, Korsakoff’s syndrome; MoCA, Montreal Cognitive Assessment; NA; not applicable; NIS, Neuropsychological Impairment Scale; RBANS, Repeatable Battery for the Assessment of Neuropsychological Status; RFFT, Ruff Figural Fluency Test; ROCF, Rey–Osterrieth complex figure; ROC, Receiver Operator Characteristic; TEDCA, TEst of Detection of Cognitive impairment in Alcoholism; TMT, Trail Making Test; WAIS, Wechsler Adult Intelligence Scale; WMS, Weschler Memory Scale.
