# Supplementary material for: Informing the development of diagnostic criteria for differential diagnosis of alcohol-related cognitive impairment (ARCI) among heavy drinkers: A systematic scoping review
Source: PLoS One. 2023 Feb 8;18(2):e0280749. doi: 10.1371/journal.pone.0280749 (PMC9907814; doi:10.1371/journal.pone.0280749)
Supplement: Attachment — Submitted filename: PLOS Feb 2022.docx [file pone.0280749.s005.docx]

You Plos One

Feb 2022

**OVERALL COMMENTS**

Thank you for the opportunity to review this interesting manuscript. This is a very important systematic review that highlights a real gap in the literature regarding this condition. It notes a paradox: we (myself included) consistently call for valid diagnostic criteria for ARBD/ARBI/ARNI but have failed to invest resources into developing them through rigorous empirical work. There are a few areas where I think the introduction/ discussion could be expanded to better account for the complexity involved in defining and diagnosing this condition, but these can be easily addressed in a revision of the paper.

Below I provide specific comments on each of the individual sections within the manuscripts.

**INTRODUCTION**

The authors choose to use the term “alcohol related brain injury”. This term is not widely used outside of very small circles in the UK and is semantically confusing/ambiguous — “injury” suggests the person has sustained a physical injury, and the term overall insinuates that somebody has experienced a brain injury that occurred whilst intoxicated, which is certainly not what the authors are referring to. I strongly suggest the authors consider alternative terms if they wish to attract international attention with this work, some of which I discuss below:

- Alcohol related brain damage - this phrase has been used in several studies published by myself and others in the UK over the last few years and is widely recognised by many clinicians in the UK, although it is admittedly not widely used outside of the UK. Further, some clinicians dislike the use of terms like “damage” and “injury” as they imply permanent physical damage. See the following paper (please note, I am referring you to this paper that I authored as I think it is relevant to this point and several others in your paper, but I do not expect you to cite the paper or discuss it in any way if you do not feel it is suitable/relevant to your work):

Heirene, R. M., John, B., O’Hanrahan, M., Angelakis, I. & Roderique-Davies, G. (2021). Professional Perspectives on Supporting Those with Alcohol-Related Neurocognitive Disorders: Challenges & Effective Treatment. *Alcoholism Treatment Quarterly*, *39*(3), 1–27. <https://doi.org/10.1080/07347324.2021.1898294>

Full text access: <https://robheirene.netlify.app/publication/heirene_et_al_2021_perspectives_on_arbd_treatment/Heirene_et_al_%282021%29_Perspectives_on_ARBD_treatment.pdf>

Alcohol-related cognitive impairment - this phrase is unambiguous, uncontroversial, and widely accepted worldwide

- alcohol-related neurocognitive impairment - again, this phrase is unambiguous, uncontroversial, and widely accepted worldwide. This term is also more semantically accurate and simply cognitive impairment”.

It is good that the authors note the other diagnostic terms used in the confusion this brings.

Third paragraph: the Smith and Hillman study referred to here only discussed recovery rates in those with Korsakoff’s syndrome. The authors appear to exclude this manifestation from ”ARBI” in their introduction, no? If they include this syndrome under their definition of ARBI then this needs to be clarified in the first paragraph or two.

Final paragraph: further to my first comment, the authors state that criteria for ARD have been adapted for a probable diagnosis of “ARBI” but Wilson actually used the term “alcohol related brain damage” in the article cited.

Given the overall purpose of this review, the authors need to better clarify what they mean by “ARBI” at the start of the manuscript. What is and isn’t included under this definition? Can you detailed description of the symptomology be provided, whilst acknowledging heterogeneity in presentation and noting this as key? It needs to be absolutely clear what “condition” is been referred to before we move on to determining how best to screen for it. Later in the article, the authors refer to a variety of different study populations as having “ARBI” including those in Brown et al. and Wester et al., but these are described differently by their respective authors. Perhaps the authors could discuss some of these studies and the samples studied in the introduction as examples of populations with ARBI according to their definition?

**METHODS**

Why was the protocol not registered in advanced if it was developed prior to conducting the review? This seems very strange and needs clarifying.

INCLUSION CRITERIA

the objectives stated here differ slightly from those stated at the end of the introduction? There is some inconsistency here.

Were reviews included? Please clarify

Was there a language restriction? Please clarify

The authors state they excluded samples “where they included mixed samples of drug and alcohol users.” Did this include samples will use both alcohol and drugs simultaneously? Please clarify. Polysubstance misuse is common and among this group.

SEARCHING FOR AND SELECTING RELEVANT STUDIES

This process of searching for and selecting articles seems scientifically valid and thorough. It is great that the authors used three reviewers to independently screen for potential studies at the full text level. At the title and abstract screening level, did the authors record the level of consistency between the two reviewers when screening the initial 20% of articles? It would be good to have some indication of interrater reliability.

DATA CHARTING PROCESS AND QUALITY ASSESSMENT

DATA SYNTHESIS

I have no other concerns or comments regarding the other subsections of the methods section—everything is clearly explained.

**RESULTS**

in the first paragraph of this section, the authors state that “20 records were kept and comprised the final set of included studies in the review”, but figure 1 shows only 19 studies were kept. Please clarify which is correct.

The authors state that only one study (32) included patients with a diagnosis of ARBI. However, our study (19; see below) included participants with a diagnosis of alcohol related brain damage, which the authors appear to include in the definition of ARBI. Please clarify whether this is because you only included studies that diagnose participants according to the ARD criteria.

19: Brown P, Heirene RM, John B, Evans JJ. Applicability of the ACE-III and RBANS cognitive tests for the detection of Alcohol-Related Brain Damage. Frontiers in psychology. 2019;10:2636.

Page 14: can the authors please provide a brief narrative summary of the results from the quality assessment process? I think it’s fine to include the full table of outcomes in supplemental information, but the readers need to have some indication of what the outcomes were here for ease of interpretation. A simple sentence or two stating what percentage of studies met each of the criteria in the assessment would suffice.

**DISCUSSION**

First paragraph: the authors provide a clear and succinct summary of the findings here and make some important inferences regarding the lack of research in this area despite calls for validation of diagnostic criteria.

Third paragraph: the varied mechanisms underlying ARBI is an important point here. I think we have a good enough understanding of the mechanisms involved to know that they are multiple and varied. As a corollary, we shouldn’t expect there to be strict diagnostic/ screening criteria that apply to every case of ARBI/ARBD/ARNI. I think the authors need to make more of a point of this and, although we need to better test existing and new criteria and cut-off scores for these, we should recognise that there will always be some degree of clinical judgement involved in making this diagnosis. It seemed like the complexity diagnosis and heterogeneity in presentation is not fully appreciated here and could be expanded upon.

The conclusions derived are appropriate and important.

**FINAL COMMENTS**

I hope the authors find the above comments useful in revising their manuscripts for publication.

Sincerely,

Rob Heirene (I sign my reviews)
