# Supplementary material for: Informing the development of diagnostic criteria for differential diagnosis of alcohol-related cognitive impairment (ARCI) among heavy drinkers: A systematic scoping review
Source: PLoS One. 2023 Feb 8;18(2):e0280749. doi: 10.1371/journal.pone.0280749 (PMC9907814; doi:10.1371/journal.pone.0280749)
Supplement: Attachment — Submitted filename: Diagnostic criteria for ARBI-Response to Reviewers.docx [file pone.0280749.s006.docx]

| **ADDITIONAL EDITOR COMMENTS:** |  | |
| --- | --- | --- |
| Thank you for submitting your paper. I am very grateful to the two expert reviewers for their thorough read of your manuscript and for their insightful suggestions. Like the reviewers, I feel the paper has its strengths and addresses an important issue, but feel it would benefit from a much stronger rationale – by explaining some of issues, limitations with the existing criteria. At the moment it lacks a clearly defined purpose and reads more of a summary of what others have done and where they overlap. By providing some context in terms of the complexity, difficulties we face in defining/diagnosing alcohol-related cognitive impairment its contribution to the literature will become a lot clearer. When revising your manuscript, I would encourage you to refer the recent systematic review by Ko et al (2021) and to look carefully at the conditions under which cognitive impairment screening tools can be used. | Thank you for you positive and constructive comments on the paper. We have reorganised and added to the Introduction to make the context and purpose of the paper clearer to the reader. | |
| **OTHER MINOR ISSUES** |  | |
| Make it clear that the statement below refers to people with Korsakoff’s, particularly since this is a criticism of the recent, similar Heirene et al. (17) review With treatment, Smith and Hillman (13) estimated that full recovery can be achieved in approximately 25% of patients, and among the remainder, 50% can achieve a partial or minor recovery | This has been clarified in the text as follows (pg 4, 1st para):  *Evidence from the broader spectrum of conditions associated with cognitive impairment suggests that recognition and a diagnosis of ARCI would significantly improve the long-term prognosis for patients. Among patients with Korsakoff’s Syndrome (KS), Smith and Hillman* | |
| The paper would benefit from starting clearer objectives. Currently aim 2 (on page 4) seems rather weak, “the identification of further work in identifying the best tools”, surely this would be a by-product of addressing aim 1 “summarising the evidence of existing tools/criteria and any validations of those”. | We have deleted the following part of the sentence to ensure that the objectives of the paper/review are clearer: *“the purpose of supporting further work to explore which of the available tools and/or operational criteria best facilitate the rapid identification of patients with suspected ARBI”.* | |
| As noted by one of the reviewers, the fact that a protocol was developed in advance (but not registered) is a major limitation and should be acknowledged. | At the time of developing the protocol, we were aware of PROSPERO, the international prospective register of systematic reviews. We planned to register the protocol with PROSPERO but found that it does not accept scoping reviews. We were not aware of other free options for prospective registration at the time of developing the protocol. We now understand that paid options are available and/or the protocols can be published on free open science platforms. We have acknowledged that the protocol was not prospectively available in the limitations. | |
| Finally with regards to the title - perhaps it needs a word like "Towards developing" or "Informing the development" as it appears rather overstated in its current form, relative to what the paper describes. | ‘Informing’ added | |
| I am confident you will find the reviewers comments helpful in the revision of the manuscript | We have. Thank you. | |
| **REVIEWER #1 COMMENTS** | | **AUTHORS RESPONSE** |
| Reviewer #1: Thank you for the opportunity to review this manuscript entitled Development of diagnostic criteria for differential diagnosis of alcohol-related brain injury among heavy drinkers: a systematic scoping review. This manuscript is a timely and useful review highlighting an important issue in the addiction field regarding the lack of criteria for diagnosing alcohol related brain injury. | | Thank you for your comments. No response needed. |
| Overall the manuscript is of an appropriate quality and followed a systematic scoping review methodology to extract key themes from the included literature. Prior to recommendation for publication, however, I would like to see the following comments addressed. | | Please see below for how individual comments have been addressed. |
| **GENERAL COMMENTS** | |  |
| Overall I missed a more in-depth critique of the currently available criteria. For instance, specifically what are the current issues or practical concerns with applying the available criteria? I think inclusion of this within the introduction and discussion would be helpful for readers in highlighting the specific issues experienced by clinicians along with some potential avenues for improvement that future studies could incorporate or explore. This would help strengthen the discussion which has only a very small section devoted to this issue before moving onto screening tools. | | Please see below under Introduction for how we have responded to the comment about the need for more information about the currently available criteria. |
| **INTRODUCTION** | |  |
| In the introduction/methods, it might be helpful for Table 1 to be expanded to include all available formal diagnostic classification systems for ARBI/ARD. This would help summarise the lay of the land for readers and provide a better context for the review.  Further to this, I think the manuscript could benefit from some additional commentary about the DSM and ICD diagnostic classification systems including any strengths and identified limitations. | | We have expanded the section on ICD and DSM systems in the second paragraph to explain why they are not used in clinical practice to diagnosis ARCI. One the main issues, which we have sought to make clearer, is that there is a shortage of rigorous empirical research done in clinical practice to establish the validity/usefulness of the new and existing criteria. |
| **METHODS** | |  |
| The systematic scoping review procedure was well executed and described. | | Thank you for your comments. No response needed. |
| Please provide a stronger justification for excluding studies that solely used DSM/ICD classifications and a statement regarding the number of studies that were excluded on this basis. | | We have clarified in the text that this exclusion was specific to ICD-10/11 and DSM-IV as neither version provides unifying criteria for ARCI.  We dropped the diagnosis terms in our revised strategy and in practice no exclusions were made because of this criterion. |
| The exclusion criteria regarding mixed samples of drug and alcohol users is a reasonable one, however, this should be acknowledged as a general limitation given the clinical reality that polysubstance use is often the norm in AOD settings rather than the exception and research studies often struggle to reflect this. | | We have added a paragraph about the exclusion of studies at the start of the results section. We acknowledge that polysubstance use is common among the patient group and did not exclude samples reporting polysubstance use.  As we did not include explicit inclusion criteria in relation to other substance use disorders, we have added the following text to clarify what types of studies were excluded (pg 7; 4^th^ para):  *Twelve studies were considered ‘near misses’ and judged to have narrowly failed to meet the inclusion criteria in respect of the review’s secondary objective as the samples included patients with diagnoses of dependence on substances other than alcohol. We agreed post hoc to exclude these studies where the sample included patients with a primary diagnosis (based on DSM or other recognised criteria) of abuse or dependence on a substance other than alcohol.*  We have acknowledged that this may be a limitation in the Discussion (pg 20, 2^nd^ para of Limitations). |
| Page 6 paragraph 3: Typo. Diagnosis should be diagnose. | | This typo has been corrected. |
| **RESULTS** | |  |
| Table 3 – Note that the MoCA now requires training certification to administer. Note also that some assessment measures have user restrictions that prevent unqualified individuals being able to purchase or administer the measure. For instance, the RBANS and NIS have a User Level B (Psychologist, Speech Pathologist, Occupational Therapist, Special Education Teacher, Human Resources Professional, Psychiatrist, Paediatrician). Check with each test publisher to determine the User level.  In highlighting these screening tools I would recommend a note of caution that they ought to be administered and interpreted by an appropriately qualified health professional with experience in the field of cognition. For instance, I note that the TEDCA personnel use is “unclear” given the lack of formal guidance by a test publisher or equivalent, however, it is clear that the measure contains several neuropsychological assessment tools that are generally recommended for use by clinical neuropsychologists and I would not consider them to fall into the category of “easy brief administration by non-specialised healthcare personal in the clinical and research field”, as was concluded by the TEDCA study authors!  A good description of the concerns associated with the use of screening tools in the AOD sector is in the second paragraph of the clinical recommendations section of the following paper: doi:10.1017/S135561772100103X.  This paper also includes an extensive review of screening tools utilised in the AOD sector and would be worth cross checking and discussing where appropriate. For instance, their conclusions are quite similar in that very few studies have validated the use of screening tools in AOD populations with the MoCA being the most commonly used. | | Thank you for the orientation to the Ko et al., paper this has usefully informed our revisions.  With respect to clinical utility, we have removed the column that refers to Personnel in Table 3, as on reflection this was not helpful with respect to the purposes of the paper and based on that (overall) this information was incomplete and partial across the different tools. |
| Table 3 includes abbreviations that were not described in the table notes: ROCFT, TMT. | | The list of abbreviations has been checked and the missing abbreviations added. |
| In the section describing the findings of studies using screening measures (page 16-17), was abstinence discussed in any of these studies? If this data is available in the included studies it may be worth including as a column in Tables 4 & 5 to aid interpretation of the findings. | | Information about abstinence was extracted and is presented in the data extraction tables and quality assessment information in the supporting information.  We have added a paragraph about the exclusion of studies at the start of the results section (based on Reviewer #2 comment) and included additional information here about abstinence. Brief information has also been added to Tables 4 and 5. |
| Did any of the included studies present information regarding comorbid diagnoses that could impact on the provision of a diagnosis of ARBI? | | Information about sample exclusions was extracted and is presented in the data extraction tables and quality assessment information in the supporting information.  We have added a paragraph about the exclusion of studies at the start of the results section (based on Reviewer #2 comment) and included additional information here about exclusions based on co-morbidity. |
| **DISCUSSION** | |  |
| The discussion may benefit from commentary on the ICD-11 now being in effect given there has been a change in the diagnostic criteria for alcohol related dementia compared with the ICD-10. Highlighting this and discussing any avenues for further development could be valuable for readers. | | We have information to the Introduction about the DSM and ICD classification systems. Although we accept the comment and agree that is could be beneficial to add further commentary in the Discussion we are have chosen not to address this comment as it would add significantly to the word count. |
| Within paragraph three could the authors provide some commentary on any observations regarding the presence of psychiatric comorbidity or other factors observed in the included studies that may impact the differential diagnosis of ARBI in this cohort? | | We have added to paragraph three, noting that confounding factors may complicate assessment of patients with suspected ARCI. |

| **REVIEWER #2 COMMENTS** | **AUTHORS RESPONSE** |
| --- | --- |
| **OVERALL COMMENTS** |  |
| Thank you for the opportunity to review this interesting manuscript. This is a very important systematic review that highlights a real gap in the literature regarding this condition. It notes a paradox: we (myself included) consistently call for valid diagnostic criteria for ARBD/ARBI/ARNI but have failed to invest resources into developing them through rigorous empirical work. There are a few areas where I think the introduction/ discussion could be expanded to better account for the complexity involved in defining and diagnosing this condition, but these can be easily addressed in a revision of the paper. | Thank you for your comments. Please see below for our responses to each comment. |
| **INTRODUCTION** |  |
| The authors choose to use the term “alcohol related brain injury”. This term is not widely used outside of very small circles in the UK and is semantically confusing/ambiguous — “injury” suggests the person has sustained a physical injury, and the term overall insinuates that somebody has experienced a brain injury that occurred whilst intoxicated, which is certainly not what the authors are referring to. I strongly suggest the authors consider alternative terms if they wish to attract international attention with this work, some of which I discuss below:   - Alcohol related brain damage - this phrase has been used in several studies published by myself and others in the UK over the last few years and is widely recognised by many clinicians in the UK, although it is admittedly not widely used outside of the UK. Further, some clinicians dislike the use of terms like “damage” and “injury” as they imply permanent physical damage. See the following paper (please note, I am referring you to this paper that I authored as I think it is relevant to this point and several others in your paper, but I do not expect you to cite the paper or discuss it in any way if you do not feel it is suitable/relevant to your work):   Heirene, R. M., John, B., O’Hanrahan, M., Angelakis, I. & Roderique-Davies, G. (2021). Professional Perspectives on Supporting Those with Alcohol-Related Neurocognitive Disorders: Challenges & Effective Treatment. *Alcoholism Treatment Quarterly*, *39*(3), 1–27. <https://doi.org/10.1080/07347324.2021.1898294>  Full text access: <https://robheirene.netlify.app/publication/heirene_et_al_2021_perspectives_on_arbd_treatment/Heirene_et_al_%282021%29_Perspectives_on_ARBD_treatment.pdf>  Alcohol-related cognitive impairment - this phrase is unambiguous, uncontroversial, and widely accepted worldwide  Alcohol-related neurocognitive impairment - again, this phrase is unambiguous, uncontroversial, and widely accepted worldwide. This term is also more semantically accurate and simply cognitive impairment”.  It is good that the authors note the other diagnostic terms used in the confusion this brings. | Thank you for providing your considerations on this issue. The authors have discussed the terminology used and we have agreed with the suggestion to change the overarching term used to ‘Alcohol Related Cognitive Impairment’.  The introduction has been reorganised to better clarify the sources of variation and how they have been used by clinicians and researchers.  Please see our responses to the next set of comments. |
| Third paragraph: the Smith and Hillman study referred to here only discussed recovery rates in those with Korsakoff’s syndrome. The authors appear to exclude this manifestation from ”ARBI” in their introduction, no? If they include this syndrome under their definition of ARBI then this needs to be clarified in the first paragraph or two. | This has been clarified in the text as follows (pg 4, 1st para):  *Evidence from the broader spectrum of conditions associated with cognitive impairment suggests that recognition and a diagnosis of ARCI would significantly improve the long-term prognosis for patients. Among patients with Korsakoff’s Syndrome (KS), Smith and Hillman* |
| Final paragraph: further to my first comment, the authors state that criteria for ARD have been adapted for a probable diagnosis of “ARBI” but Wilson actually used the term “alcohol related brain damage” in the article cited. | We have clarified this by changing to the phrasing used in the original paper: *‘probable diagnosis of alcohol-related brain damage’*. |
| Given the overall purpose of this review, the authors need to better clarify what they mean by “ARBI” at the start of the manuscript. What is and isn’t included under this definition? Can you detailed description of the symptomology be provided, whilst acknowledging heterogeneity in presentation and noting this as key? It needs to be absolutely clear what “condition” is been referred to before we move on to determining how best to screen for it. Later in the article, the authors refer to a variety of different study populations as having “ARBI” including those in Brown et al. and Wester et al., but these are described differently by their respective authors. Perhaps the authors could discuss some of these studies and the samples studied in the introduction as examples of populations with ARBI according to their definition? | As noted above, we have reorganised the opening paragraphs of the Introduction. This now establishes that alongside the use of variant terms, that as umbrella terms, they have also been used to both encompass and exclude WKS.  We have added the following text as clarification about the focus of the scoping review (pg 3, end of 1^st^ para):  *For the purposes of this scoping review, we have used the term Alcohol-Related Cognitive Impairment (ARCI) and applied it in its broadest sense. We have included articles following either application of the terminology, for example, by recognising that ARD and ARCI may be used to refer to the same suspected condition, and that umbrella terms such as ARBD may either encompass or exclude WKS.* |
| **METHODS** |  |
| Why was the protocol not registered in advanced if it was developed prior to conducting the review? This seems very strange and needs clarifying. | At the time of developing the protocol, we were aware of PROSPERO, the international prospective register of systematic reviews. We planned to register the protocol with PROSPERO but it does not accept scoping reviews. We were not aware of other free options for prospective registration at the time of developing the protocol. We now understand that paid options were available or free open science platforms. |
| *INCLUSION CRITERIA* |  |
| the objectives stated here differ slightly from those stated at the end of the introduction? There is some inconsistency here. | We have checked the text for consistency between the end of the Introduction and the Inclusion criteria. We have amended the text to make it clearer that there were different selection criteria applied in respect of the two objectives for the review. |
| Were reviews included? Please clarify | Reviews were not included and we have clarified that original, primary research were eligible. |
| Was there a language restriction? Please clarify | We have made it clear that only studies published in English were eligible. |
| The authors state they excluded samples “where they included mixed samples of drug and alcohol users.” Did this include samples will use both alcohol and drugs simultaneously? Please clarify. Polysubstance misuse is common and among this group. | We have added a paragraph about the exclusion of studies at the start of the results section. We acknowledge that polysubstance use is common among the patient group and did not exclude samples reporting polysubstance use.  As we did not include explicit inclusion criteria in relation to other substance use disorders, we have added the following text to clarify what types of studies were excluded (pg 7; 4^th^ para):  *Twelve studies were considered ‘near misses’ and judged to have narrowly failed to meet the inclusion criteria in respect of the review’s secondary objective as the samples included patients with diagnoses of dependence on substances other than alcohol. We agreed post hoc to exclude these studies where the sample included patients with a primary diagnosis (based on DSM or other recognised criteria) of abuse or dependence on a substance other than alcohol.*  We have acknowledged that this may be a limitation in the Discussion (pg 20, 2^nd^ para of Limitations). |
| *SEARCHING FOR AND SELECTING RELEVANT STUDIES* |  |
| This process of searching for and selecting articles seems scientifically valid and thorough. It is great that the authors used three reviewers to independently screen for potential studies at the full text level. At the title and abstract screening level, did the authors record the level of consistency between the two reviewers when screening the initial 20% of articles? It would be good to have some indication of interrater reliability. | Interrater reliability was not recorded. Because of the wide use of terminology in the field it was difficult on occasion to discern from the titles/abstracts whether a study contained relevant information or not, so a level of disagreement was expected in the dual screening process. The discussions about disagreements were therefore used to develop confidence in the selection process conducted by the single reviewer.  The following text has been added (pg 6, 2^nd^ para):  *Interrater reliability was not recorded as a level of disagreement was anticipated based on the wide use of terminology in the field. Discrepancies in study selection were discussed and resolved and used to develop confidence in the screening of the remaining titles and abstracts which was carried out by a single reviewer (LJ).* |
| **RESULTS** |  |
| in the first paragraph of this section, the authors state that “20 records were kept and comprised the final set of included studies in the review”, but figure 1 shows only 19 studies were kept. Please clarify which is correct. | The correct number of included studies is 19. The flowchart has been corrected for the errors that have crept into the final number of included studies and reasons for full-text exclusions. |
| The authors state that only one study (32) included patients with a diagnosis of ARBI. However, our study (19; see below) included participants with a diagnosis of alcohol related brain damage, which the authors appear to include in the definition of ARBI. Please clarify whether this is because you only included studies that diagnose participants according to the ARD criteria. | We have clarified in the text that this description applies to the six studies that were descriptive reports. Reference 19 is not referred to within this set of studies and is reported in the sentences preceding as examining the diagnostic criteria for ARBI (now referred to as ARCI). |
| Page 14: can the authors please provide a brief narrative summary of the results from the quality assessment process? I think it’s fine to include the full table of outcomes in supplemental information, but the readers need to have some indication of what the outcomes were here for ease of interpretation. A simple sentence or two stating what percentage of studies met each of the criteria in the assessment would suffice. | A short paragraph has been added about quality assessment (pg 14, 1^st^ para):  *All seven studies provided adequate descriptions of the included patient samples, but exclusion based on comorbid or confounding conditions was not clear or not reported in two studies (20, 27). Further, effect sizes were only reported in two of six studies where this was deemed appropriate (19, 31). As shown in Table 4, studies typically included small to moderate sample sizes. Four studies (19, 20, 28, 34) reported clearly that they had adjusted for the risk of type 1 error in their analyses.* |
| **DISCUSSION** |  |
| First paragraph: the authors provide a clear and succinct summary of the findings here and make some important inferences regarding the lack of research in this area despite calls for validation of diagnostic criteria. | Thank you for your comment. No response required. |
| Third paragraph: the varied mechanisms underlying ARBI is an important point here. I think we have a good enough understanding of the mechanisms involved to know that they are multiple and varied. As a corollary, we shouldn’t expect there to be strict diagnostic/ screening criteria that apply to every case of ARBI/ARBD/ARNI. I think the authors need to make more of a point of this and, although we need to better test existing and new criteria and cut-off scores for these, we should recognise that there will always be some degree of clinical judgement involved in making this diagnosis. It seemed like the complexity diagnosis and heterogeneity in presentation is not fully appreciated here and could be expanded upon. | We have added the additional considerations to the Discussion (pg 19, 2^nd^ para):  *However, evaluation of the practical application of new or existing ARCI criteria, either through formal research or routine monitoring, is needed to confirm that they have utility in clinical practice and that healthcare professionals do not face barriers to their use. Further, patients with ARCI report experiencing a ‘double stigma’ within healthcare settings (10, 40) and negative attitudes towards people with problems with alcohol or other substances among healthcare professionals are known to undermine access to diagnosis, treatment, and successful health outcomes (41, 42).* |
| The conclusions derived are appropriate and important. | Thank you for your comment. No response required. |
