# Supplementary material for: Informing the development of diagnostic criteria for differential diagnosis of alcohol-related cognitive impairment (ARCI) among heavy drinkers: A systematic scoping review
Source: PLoS One. 2023 Feb 8;18(2):e0280749. doi: 10.1371/journal.pone.0280749 (PMC9907814; doi:10.1371/journal.pone.0280749)
Supplement: Attachment — Submitted filename: Diagnostic criteria for ARBI-Response to Reviewers.docx [file pone.0280749.s007.docx]

| **ADDITIONAL EDITOR COMMENTS:** |  |
| --- | --- |
| I agree with reviewer 1 that the paper would be strengthened with the addition of just a few sentences explaining how and why there were adaptations to the criteria. | We have revised the first paragraph of the Discussion to incorporate a few sentences on this matter. Please see our response to Reviewer #1 for further details. |
| **REVIEWER #1 COMMENTS** | **AUTHORS RESPONSE** |
| Thank you for the opportunity to review this revised manuscript and I thank the authors for addressing the comments with some excellent additions to the manuscript.  I have some minor comments that the authors and editor may wish to consider prior to publication. | Thank you for your comments. Please see below for our responses to each comment. |
| **Discussion** |  |
| I recognise this is a challenging point to address so it may not feasible, however, I still think the discussion could benefit from some elaboration on the available criteria and any emerging themes or issues from the included studies to help advance our thinking and maybe guide some next steps. For instance, a few of the included studies made adaptions to existing criteria, so what were these adaptions? Where they similar or not? Why did these authors feel the need to made such adaptions? | We have reorganised the text in the first paragraph and added additional information about the lack of clarity on how and why adaptations were made to the criteria. The eight papers were read through again to check for any missed details but as we have noted in this revision, there is no discussion across the studies on this matter.  *“Although the included studies reported information about the diagnostic or screening criteria used to identify suspected ARCI, except for Oslin and Cary [30], they did not have a primary aim of exploring modifications to existing criteria or present new criteria. There was a lack of discussion across the studies about how and why adaptations were made, and this limits our understanding of any emerging themes or issues that could have been gathered in the context of carrying out this review.”* |
| Another example worth raising is highlighting how disparate the criteria are vs rates of abstinence reported in the screening studies. Your description of the abstinence rates in the results is very illuminating because it shows many studies don’t adhere to the criteria, if indeed they even report abstinence rates in the first place! This is a worthy theme regarding any future criteria for clinicians and researchers to think about given the limitations in existing research and the practicalities of having patients maintain abstinence long enough to this meet criterion. | We have added additional text to the end of the second paragraph of the Discussion. Again we highlight that there were missed opportunities to learn from the currently available evidence.  *“As highlighted by Heirene et al. [10], another important area is decisions about when tools should be administered. Neuropsychological functioning improves with the duration of abstinence [43] and for patients at-risk of ARCI who present in acute care, screening must take place “within a very small window of time” [46]. Many studies included in this review did not report abstinence rates among their samples, and this was consequently another area from which we are not able garner much from the currently available research.”* |
| **Minor:** |  |
| As a reader there are a lot of abbreviations to keep track of and this risks adding to the already slightly confusing nature of all the different terms for alcohol related cognitive impairment. I would suggest considering a quick review of which abbreviations are absolutely necessary and which ones would read better in full. I realise this might add to the word count but I think for ease of reading it would be worthwhile. | We have reviewed the use of abbreviations throughout the manuscript and minimised unnecessary use of abbreviations where possible. |
| Results (page 15): I was a little lost with the revised sentence following mention of Table S4 referring to “The tool from the review by Heirene…”. I was trying to understand “what tool” so maybe this paragraph just needs a slight restructure or framing for the reader to link the previous sentence with the next. | We have edited this sentence to remove the unnecessary reference to the tool. |
| **REVIEWER #2 COMMENTS** | **AUTHORS RESPONSE** |
| The authors have appropriately revised their manuscript according to the comments provided. I have no further concerns. | Thank you for your further consideration of our manuscript. No response needed. |
